# Immobilization of *β*-CD on a Hyper-Crosslinked Polymer for the Enhanced Removal of Amines from Aqueous Solutions

**DOI:** 10.3390/polym12071620

**Published:** 2020-07-21

**Authors:** Zujin Yang, Guifang Wu, Qiuru Li, Hongxia Ai, Xingdong Yao, Hongbing Ji

**Affiliations:** 1 School of Chemical Engineering and Technology, Sun Yat-Sen University, Zhuhai 519082, China; Wugf5@mail2.sysu.edu.cn (G.W.); Liqr@mail2.sysu.edu.cn (Q.L.); 2 School of Chemical Engineering, Huizhou Research Institute of Sun Yat-Sen University, Huizhou 516216, China; 3 Fine Chemical Industry Research Institute, The Key Laboratory of Low-carbon Chemistry & Energy Conservation of Guangdong Province, School of Chemistry, Sun Yat-Sen University, Guangzhou 510275, China; aihongxia@mail.sysu.edu.cn; 4 The Key laboratory of Forest Chemistry & Engineering of Guangxi, Guangxi University for Nationalities, Nanning 210000, China; yaoxingdong@gxun.edu.cn; 5 School of Chemical Engineering, Guangdong University of Petrochemical Technology, Maoming 525000, China

**Keywords:** CDM-HCP, adsorption, amines, hyper-crosslinked polymer, interaction mechanism

## Abstract

In this paper, we adopted a simple and efficient strategy to prepare a *β*-cyclodextrin (*β*-CD)-modified hyper-crosslinked polymer (CDM-HCP). The structures and physicochemical properties of the as-synthesized polymer were also evaluated. It was applied to the removal of anilines from aqueous solutions. The introduction of *β*-CD into the hyper-crosslinked polymer significantly enhanced adsorption properties for the removal of various amines. The adsorption kinetics agreed with the pseudo-second-order mode very well. The adsorption isotherm data of *p*-methylaniline (*p*-MA) and *p*-aminobenzoic acid (*p*-ABC) were in agreement with the Langmuir isotherm, whereas aniline and *p*-chloroaniline (*p*-CA) were fitted best with the Freundlich model. The maximum adsorption capacities (*q*_max_) determined by adsorption isotherms were 148.97 mg/g for aniline, 198.45 mg/g for *p*-MA, 293.71 mg/g for *p*-CA, and 622.91 mg/g for *p*-ABC, respectively. It had higher adsorption capacities than those of some commercial polymeric resins, such as XAD-4, PA66, and AB-8. The interaction mechanism was investigated by FTIR, XPS, and the ONIOM2 method. A CDM-HCP can be regenerated efficiently and used repeatedly, indicating its potential technological applications in removing organic pollutants from actual industrial effluents.

## 1. Introduction

In recent years, the development of industrialization and urbanization has aroused global attention to environmental pollution. Organic pollutants in most developing countries have no effective treatment and are directly discharged into the environment during the production process, including the petroleum, chemical, pharmaceutical, textile, and plastics industries. Health and environmental issues have stemmed from them because of their high toxicity and difficult degradation [1,2]. Anilines are applied as intermediates in the production of pesticides, drugs, dyes, rubber, and herbicides [3,4]. Anilines are one kind of significant organic pollutants in our environment, and the discharge of these contaminants may cause great harm to the environment and the drinking water even when they exist as a trace [5]. For example, the US Environmental Protection Agency (EPA) suggests that the maximum acceptable concentration of aniline in water is 0.262 ppm [6]. For the sustainable development of the water environment, it is of significance to develop efficient ways for the removal of toxic organic substances in wastewaters. Various treatment methods such as biodegradation, photocatalytic degradation, ozonation oxidation, and adsorption have been employed to eliminate anilines from wastewaters [7,8,9]. Among these methods, adsorption is one of the most effective approaches to remove these pollutants because it has certain advantages, such as low cost, simplicity, and effectiveness.

A hyper-crosslinked polymer (HCP) is an efficient adsorbent for the removal of anilines because of specific surface areas, porous structures, and surface physicochemical characteristics [10,11,12]. The Friedel–Crafts reaction has been used to synthesize HCPs by using low crosslinked chloromethylated polystyrene (CM-PS) as a starting skeleton in the presence of Lewis acids in solution. A previous publication has shown that the as-synthesized HCP shows low equilibrium capacity for the polar compounds due to hydrophobic surface [13]. Chemical modification has been considered to introduce different functional groups such as -OH, -NH_2_, and -COOH into the polymers, which can increase the adsorption capacity for poisonous organic chemicals. Therefore, HCPs modified with polar components have been studied during the past decades [11,12,14,15].

Cyclodextrins (CDs), which include 6–8 α-(1-4)-linked *α*-D-glucopyranose units to form a hydrophobic center and hydrophilic edge, are a class of natural cyclic oligosaccharides derived from starch (named *α*-, *β*-, and *γ*-CD, respectively) [16,17]. CDs can form inclusion complexes via the weak interactions with aromatic compounds in aqueous solutions [18,19]. *β*-CD is one of the cheapest and most extensively used CDs, and it is conducive to the removal of polar aromatic compounds from wastewaters through the host–guest interactions between adsorbent and adsorbate. However, there is an obvious deficiency in the application of *β*-CD due to its solubility, and immobilization on certain supports to realize its recyclability is an ideal method. *β*-CD-based materials have been applied in removing organic pollutants such as aromatics or phenolics from the environment [20,21]. Li et al. [22] constructed a hyper-crosslinked *β*-CD porous polymer by crosslinking benzylated *β*-CD with dichloroxylene via a Friedel–Crafts reaction, which has a high surface area and good thermal stability with an adsorption capacity of 278 mg/g for bisphenol A at 25 °C. Dai et al. [23] also synthesized a *β*-CD-based HCP by reacting benzylated *β*-CD with formaldehyde dimethyl acetal, and an exceptional adsorption performance was observed for the removal of aromatic compounds in aqueous solutions. However, their industrial applications have been limited by the complex preparation process and the use of large organic solvents. Developing an adsorbent with high efficiency and low cost is urgent for the implementation of this process.

**Figure 1:**
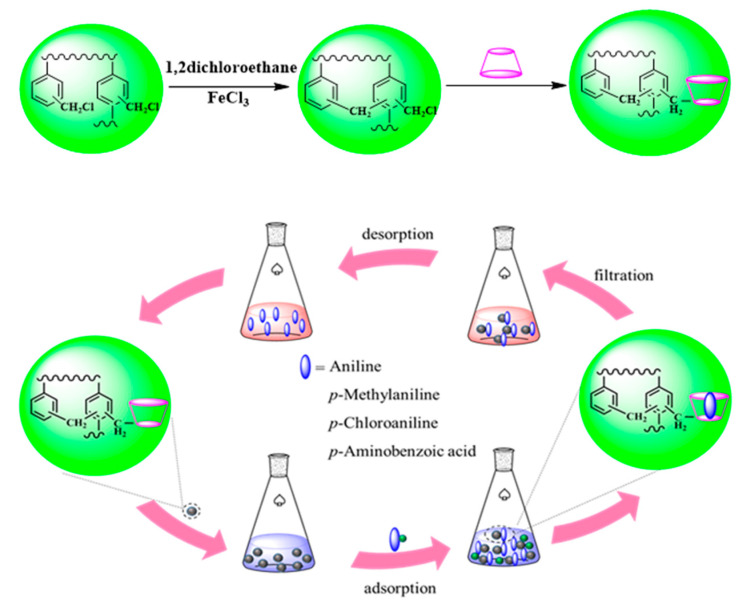
Schematic presentation for the synthesis of a *β*-cyclodextrin-modified hyper-crosslinked polymer (CDM-HCP) and the adsorption process of amines.

In this work, we first used a facile Friedel–Crafts alkylation reaction to synthesize a *β*-CD-based hyper-crosslinked polymer (CDM-HCP) (Figure 1). The structure analysis of the as-synthesized CDM-HCP was carried out with some structural characterization, and the CDM-HCP showed strong preferential adsorption toward anilines from water. Adsorption kinetics and aniline isotherms onto the CDM-HCP were also tested. The interaction mechanism between adsorbent and adsorbate was also proposed based on XPS, FTIR analyses, and quantum chemical calculations.

## 2. Materials and Methods

### 2.1. Chemicals

*β*-Cyclodextrin (*β*-CD, purity > 99%) was supplied from Shanghai Boao Biotechnology (Shanghai, China). Chloromethylated polystyrene (CM-PS) beads (crosslinked with 8% divinylbenzene (DVB), 18% chlorine content) were obtained from Nankai Chemical Co., Ltd. (Tianjin, China). All the other reagents used were at least of analytical grade. Distilled water was used from the Millipore water purification system.

### 2.2. Synthesis of the CDM-HCP

As shown in Figure 1, the CDM-HCP was prepared based on a previous synthesis procedure [24]. First, a 10 g portion of CM-PS beads was put into 50 mL of 1,2-dichloroethane (DCE) to soak overnight. Under mechanical stirring, 1.2 g of FeCl_3_ (-CH_2_Cl: FeCl_3_ = 1:1) was completely dissolved in the reaction mixture. Then, the solution was heated to 115 °C and kept at the same temperature for 12 h under N_2_ protection. After being cooled to 25 °C, the CDM-HCP was separated and washed with 1% aqueous hydrochloric acid (*w*/*w*) and distilled water, respectively, until pH was neutral. After the reaction, the CDM-HCP was extracted in a Soxhlet extractor (Shanghai Hongji Instrument Co., Ltd. Shanghai, China) using ethanol as solvent for 24 h. Finally, the hyper-crosslinked polymer (HCP) was dried in a vacuum oven at 80 °C for 12 h.

To prepare the CDM-HCP, 1.0 g of *β*-CD and 25 mL N′N-dimethyl formamide with the appropriate amount of organic base were put into a 50 mL round-bottomed flask. Then, a 2.5 g portion of dry HCP was swollen in the mixture, heated to 90 °C, and kept at the same temperature for 12 h. Finally, the resulting CDM-HCP was filtered, washed with deionized water and MeOH, and dried under vacuum at 90 °C for 6 h to obtain the product.

### 2.3. Adsorbent Characterization

As described in a previously reported study [25], an ion chromatographic method was used to determine the chlorine content in the polymers. pH values were obtained by using a PHS-3C pH meter (Inesa, Beijing, China). The structure of the polymers was analyzed with a TENSOR 37 Fourier transform spectrometer (Bruker, Ettlingen, Germany). A Netzsch STA-449C thermal analysis system (Netzsch Corporation, Selb, Germany) was used to calculate the weight loss of the CDM-HCP. A specific surface area and pore size analyzer (Micromeritics Instrument Corporation, Norcross, GA, USA) was applied to measure the Brunauer–Emmett–Teller (BET) surface area and pore distribution of the polymers. XPS spectra (Thermo, Pleasanton, CA, USA) were recorded on an ESCALAB 250XI X-ray photoelectron spectroscope equipped with X-ray monochromatization (ESCALAB 250, Thermo Electron, Altrincham, UK). Surface properties of the polymers in aqueous phase were detected by a contact angle measuring system (OCA20, DataPhysics, Filderstadt, Germany).

### 2.4. Adsorption Experiments

Adsorption properties of the CDM-HCP for anilines were performed at the initial pH 7.0 for each aniline. A 0.1 g portion of the CDM-HCP was put into 100 mL of aniline solution with an initial concentration of 1000 mg/L. The adsorption reached equilibrium at a constant speed of 200 rpm at 20 °C, and the CDM-HCP was separated by vacuum filtration. The final concentration of anilines was analyzed using a UV-Vis spectrometer (Shimadzu UV-2450, Kyoto, Japan), and the adsorption capacity of the anilines was calculated as follows:

(1) $$q_{e}=\frac{(C_{i}-C_{e})V_{0}}{m}$$

where *q_e_* is the amount of anilines adsorbed per g of adsorbent (mg/g); *C_i_* is the initial concentration of anilines (mg/L); *C_e_* is the equilibrium concentration of anilines in solution (mg/L), *V*_0_ is the volume of the initial solution (mL); and *m* is the dry weight of the CDM-HCP (g). We further studied the effect of different *β*-CD contents (0–80 μmol/g), ionic strength (C_NaCl_ = 0–0.5 mol/L), pH (3.0–11.0), adsorption kinetics (0–800 min), and adsorption isotherms (100–1000 mg/L) of anilines at different temperatures.

### 2.5. Desorption and Regeneration Experiments

The method of desorption and regeneration experiments was carried out according to previous studies [26,27]. When the adsorption attained saturation, the CDM-HCP was desorbed with MeOH and water, respectively, to remove residual MeOH. In all the desorption tests, the tested samples were measured using a UV-Vis spectrometer. The gel adsorbent was reused five times under similar adsorption conditions. The concentration of residual anilines in the filtrate was determined, and the adsorption capacity of each aniline at an interval were calculated with Equation (1).

### 2.6. Computational Chemistry Calculations

**Figure 2:**
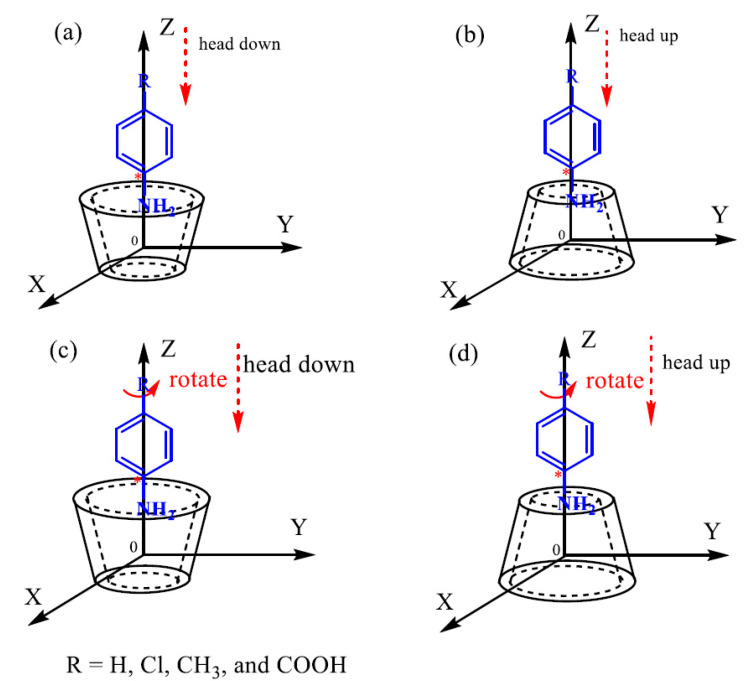
Inclusion processes of *β*-CD with guests: (**a**) head down, (**b**) head up of *β*-CD with anilines for steps; (**c**) head down, (**d**) head up of *β*-CD with anilines for dihedral angles.

The inclusion formation of the CDM-HCP and anilines is shown in Figure 2. Aniline, *p*-MA, *p*-CA, and *P*-ABC are included by *β*-CD moiety in the CDM-HCP, and the adsorption performance of the CDM-HCP is not different from the property of the *β*-CD molecule, which is in agreement with previous studies [28,29,30]. The Gaussian 09 software package (Gaussian, Inc., Wallingford, CT, USA) was used to perform these theoretical calculations, which are shown in the Supplementary Materials (Computational methods).

## 3. Results and Discussion

### 3.1. Characterization of the CDM-HCP

**Figure 3:**
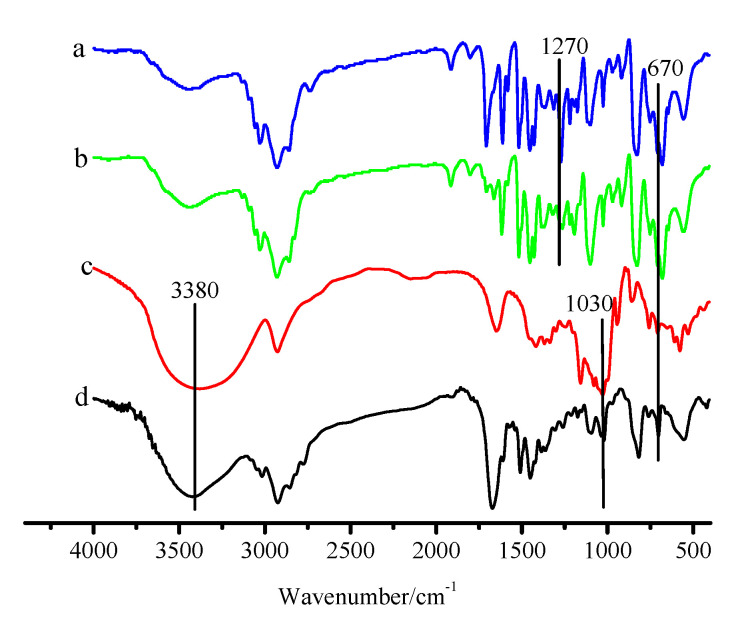
FTIR of crosslinked chloromethylated polystyrene (CM-PS) (**a**), HCP (**b**), *β*-CD (**c**), and CDM-HCP (**d**).

The CDM-HCP was obtained by the reaction of the swollen CM-PS with *β*-CD in DCE in the presence of anhydrous FeCl_3_. SEM images of the CM-PS and CDM-HCP show that CM-PS has a smooth spherical surface. After being modified by *β*-CD, the CDM-HCP still retains spherical structure. However, its surface becomes rough and large crystalline particles of rather irregular size were observed on the surface of the CDM-HCP (Figure S1 (Supplementary Materials)). Figure 3 shows that the characteristic bands at 1270 and 670 cm^−1^ are due to the C-Cl stretching vibration of the CM-PS’s CH_2_Cl groups (Figure 3a) [31]. After the Friedel–Crafts alkylation reaction, the adsorption peak at 1270 cm^−1^ is significantly weakened for HCP (Figure 3b). In addition, the CM-PS’s chlorine content decreases from 18.01% to 4.32% (HCP). The results indicate that the CM-PS’s C-Cl groups could be involved in the reaction and transformed to -CH_2_- groups. The results are in agreement with previous studies [32,33]. The bands at 3380 and 1030 cm^−1^ are due to -OH and C-O groups of *β*-CD [34] (Figure 3c). After the reaction of the HCP with *β*-CD, the band at 1030 cm^−1^ appears in the CDM-HCP’s FTIR spectrum, and the bands at 1270 and 670 cm^−1^ nearly disappear, implying that the residual chlorine on the HCP’s surface could be substituted by *β*-CD. In addition, the HCP’s chlorine content further decreases to 0.24% and the adsorption band 3380 cm^−1^ was increased by increasing the content of *β*-CD (Figure 3d), suggesting that *β*-CD exited in HCP [33]. In addition, Figure S2 (Supplementary Materials) shows the XPS wide scan of the HCP and CDM-HCP, respectively. In comparison with the HCP, the O_1s_ peak of the CDM-HCP was observed at 532.50 eV, whereas the Cl_2p_ peak of the HCP nearly disappeared. For the C1s spectrum of the CDM-HCP in Figure S2 (Supplementary Materials), different valence states of carbon were present on the HCP’s surface, and the peaks at 284.7 and 285.1 eV were due to the presence of C = C and C-O of *β*-CD, respectively, indicating the successful immobilization of *β*-CD in HCP [35]. 

**Figure 4:**
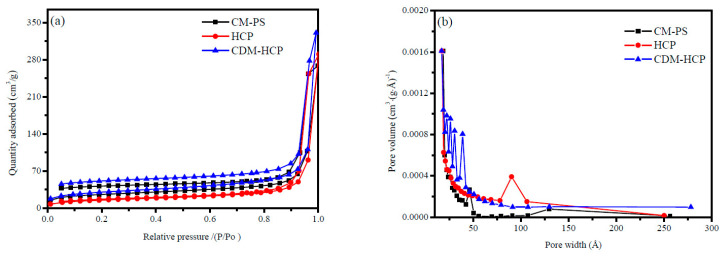
Adsorption–desorption isotherms (**a**) of N_2_ at 77 K and pore-size distribution (**b**) of CM-PS, HCP, and CDM-HCP.

Further analysis by TG (Figure S3 (Supplementary Materials)) showed that the CDM-HCP has good thermal stability with a thermal decomposition temperature of 375–400 °C due to its highly crosslinked network [36]. Such a high thermal stability is very conducive to adsorption and regeneration of the CDM-HCP in industrial applications. Figure 4 illustrates the N_2_ adsorption–desorption isotherms and pore-size distribution for the CDM-HCP. The raw material CM-PS is nonporous when it is in a dry state, but it could produce macropores after swelling in DCE, making the -CH_2_Cl groups to be extended completely. It is attributed that they would have an electrophilic substitution with the neighboring benzene rings, inducing abundant rigid methylene crosslinking bridges, which would produce these macropores after drying (Figure 4a). The results indicate that the Friedel–Crafts reaction could result in higher BET surface area and pore volume. After adding *β*-CD to the reaction, the N_2_ capacity decreases quickly, implying that the BET surface area decreases and some micropores mostly in the range of 20–50 nm exist in the CDM-HCP (Figure S2b and Table S1 (Supplementary Materials)). This result indicates that the HCP’s surface pores are turned into micropores with the addition of functional groups [37]. Table S1 (Supplementary Materials) indicates that the O content of the CDM-HCP is 6.79%, which may be due to the introduction of *β*-CD, resulting in an increase in oxygen functional groups. In addition, its lower contact angle (108°) was observed in comparison to that of the HCP (142°). The results show that the addition of *β*-CD into the HCP increases its hydrophilicity, implying that the −OH groups of *β*-CD are uploaded onto the surface of the hole in the HCP.

### 3.2. Adsorption Performance of the CDM-HCP

**Figure 5:**
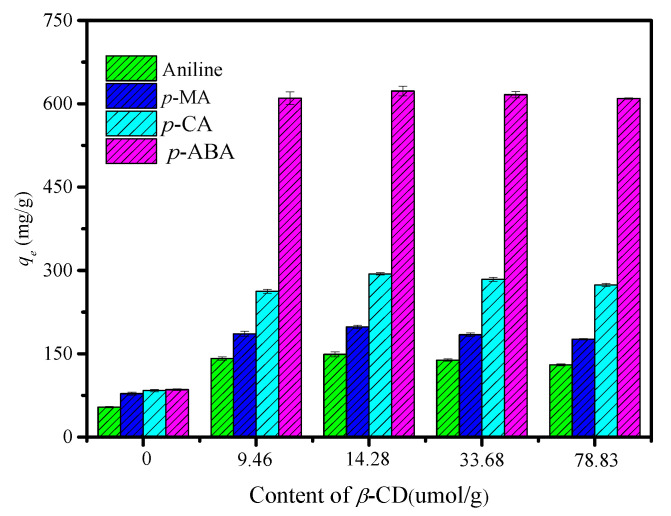
Effect of the amounts of *β*-CD on the aniline adsorption of the CDM-HCP.

Figure 5 shows that *q_e_* was increased with increasing amounts of *β*-CD up to 14.28 μmol·g^−1^. However, *q_e_* decreased when the amounts of *β*-CD exceeded 14.28 μmol·g^−1^. It was due to the high *β*-CD loading amounts, which led to the non-uniformity of the particle size distribution of the CDM-HCP, decreasing the mass transfer of the anilines.

**Figure 6:**
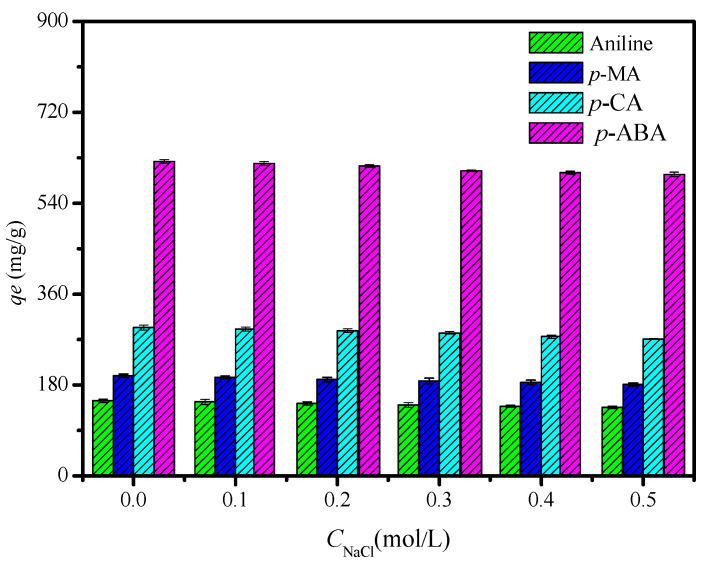
Influence of NaCl concentration on aniline removal using the CDM-HCP as adsorbent.

Figure 6 shows that the changes in ionic strength had no significant effects on the adsorption of anilines, demonstrating that the CDM-HCP has high stability within a wide range of ionic strength. *q_e_* slightly decreased with increasing NaCl concentration in the range of 0.1 to 0.5 mol·L^−1^. It may be that the solubility of adsorbate will increase significantly with increasing electrolyte concentration due to the salt effect, weakening the adsorption capacity of the adsorbent.

**Figure 7:**
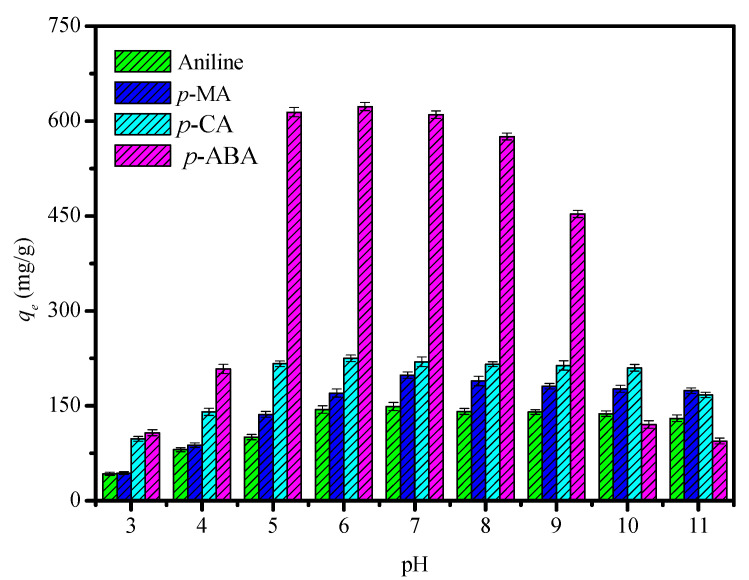
Effect of pH on the adsorption of anilines by the CDM-HCP.

The solution’s pH is an important factor affecting the adsorption of anilines on the CDM-HCP. The influence of pH on the aniline adsorption by the CDM-HCP was studied in a pH range of 3 to 11. Aniline adsorption was obviously affected by the solution’s pH, which affected the surface charge of the adsorbent and the ionization degree and speciation of the adsorbate. As shown in Figure 7, when the solution’s pH was less than or equal to 4, *q_e_* for the anilines in the aqueous phase was extremely low, which was probably because the cyclodextrin molecules were hydrolyzed under acidic conditions and their structure was destroyed, decreasing the adsorption capacity. When pH was from 5 to 7, the adsorption capacity of the CDM-HCP rose significantly for the anilines. In this case, the adsorbate existed in a molecular state, which facilitates the interaction with the functional groups of the CDM-HCP and had a maximum *q_e_* of about pH 7. However, higher solution pH also decreased the uptake of anilines. It may be due to the formation of soluble hydroxyl complexes by decreasing the free ions. The optimum pH values were 7.0 for aniline, 7.0 for *p*-MA, 6.0 for *p*-CA, and 6.0 for *p*-ABC.

### 3.3. Adsorption Kinetics

**Figure 8:**
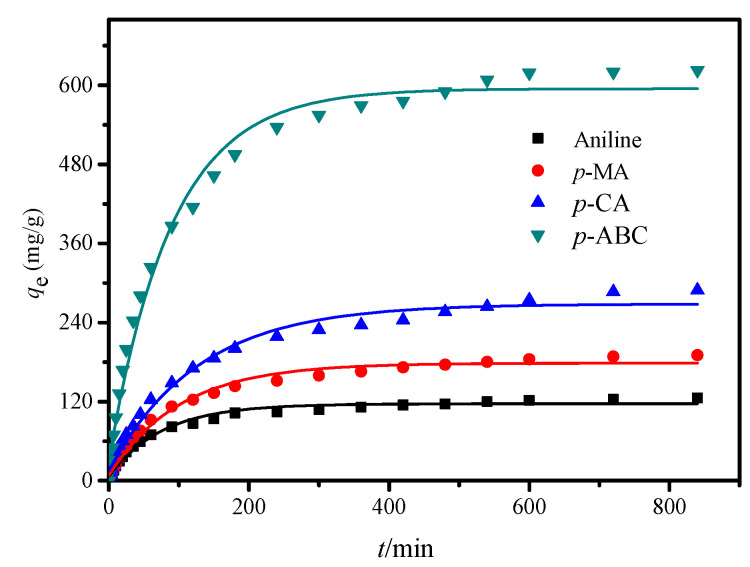
Adsorption kinetics of anilines on the CDM-HCP.

Figure 8 displays adsorption kinetic curves of anilines on the CDM-HCP versus the contact time at 20 °C. As shown in Figure 8, *q_e_* increased significantly within 200 min and then increased slowly in the subsequent step until reaching equilibrium after 600 min. A further increase of contact time was insignificant for the equilibrium *q_e_* of adsorbate. This may be due to the fact that the active adsorption sites were sufficient at the beginning of adsorption, and decreased with the increment of adsorption time. Therefore, the adsorption time of 600 min was sufficient to attain equilibrium for the CDM-HCP in the aniline adsorption, which was selected for the further experiments.

The fit of the experimental data to the pseudo-first-order, pseudo-second-order, and intraparticle diffusion models for the CDM-HCP was performed in Figure S4, and the calculated kinetic parameters are summarized in Table 1.

The results indicated that the correlation coefficient of the pseudo-second-order kinetic model (*R*^2^ > 0.99) was higher than that of the pseudo-first-order kinetic model (*R*^2^ < 0.99), implying that the adsorption process was more consistent with the second-order kinetic model. The results showed that the adsorption process may involve the mechanism of chemisorption. Furthermore, the theoretical calculated values (*q*_e,cal_) obtained from the pseudo-second-order model were in good agreement with the experimental values (*q*_e,exp_), indicating the validity of the model to the adsorption system. According to the intraparticle diffusion model, *q_t_* to *t*^1/2^ is not a straight line through the origin, showing that internal diffusion is not the mail-limiting mechanism for the aniline adsorption. Adsorption of anilines onto the CDM-HCP is divided into three steps as follows: diffusion of anilines to the external surface of the CDM-HCP; diffusion of anilines into the pores of the CDM-HCP; and adsorption of anilines onto the active sites of the CDM-HCP. Similar results have been reported for the adsorption of organic pollutants [38].

### 3.4. Adsorption Isotherm

**Figure 9:**
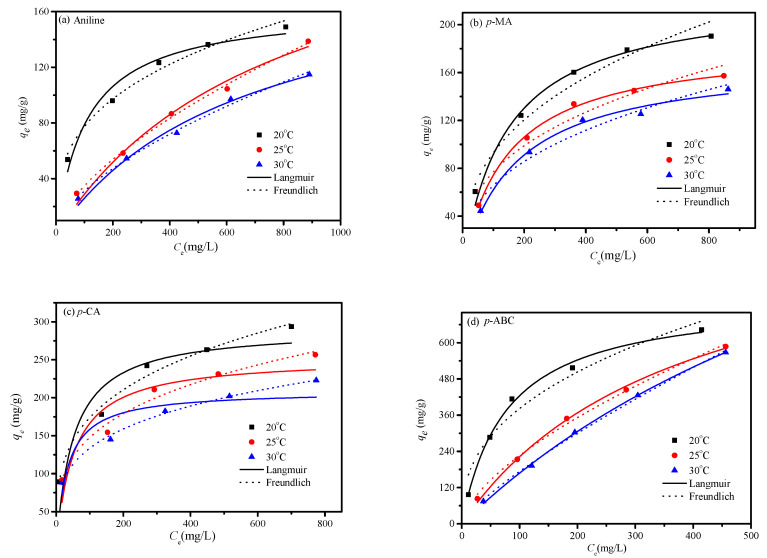
Isotherm model curves for the adsorption of aniline (**a**), *p*-MA (**b**), *p*-CA (**c**) and *p*-ABC (**d**) on the CDM-HCP; solid line is the Langmuir model and dashed line is the Freundlich model.

An adsorption isotherm can be used to clarify the interaction mechanism between adsorbent and adsorbate. Langmuir, Freundlich, and Dubinin–Radushkevich (D–R) models were applied to study the saturated adsorption behavior and capacity (Supplementary Materials). Adsorption isotherms of anilines and initial aniline concentrations in the ranges of 100–1000 mg/L at 20, 25, and 30 °C are displayed in Figure 9. The isotherm parameters and correlation coefficient (*R*^2^) are listed in Table 2.

The adsorption equilibrium was significantly affected by the initial concentrations of anilines at the different temperatures. *q_e_* quickly increased with the increase of initial concentrations until reaching equilibrium. In addition, *q_e_* onto the CDM-HCP obviously decreased with rising temperature, implying that this is of an exothermic nature [39]. The *q*_max_ (mg) values of aniline, *p*-MA, *p*-CA, and *P*-ABC calculated from the Langmuir model were 163.25, 225.44, 291.50, and 752.81 mg/g at 20 °C, respectively (Table 2). From *R*^2^, the Langmuir isotherm model demonstrated a better application to the adsorption of *p*-MA and *p*-ABC onto the CDM-HCP, indicating their anchoring to the functional groups of the CDM-HCP with the formation of monolayer surface coverage. In contrast, aniline and *p*-CA obeyed the Freundlich isotherm model better, indicating that this model was used to characterize the equilibrium adsorption with multilayer adsorption. Previous results indicated that if 1 < *n* < 10, the adsorption can occur smoothly, it is difficult to take place if 0.5 < *n* < 1, and it cannot perform for *n* < 0.5 for the Freundlich equation [40]. From Table 2, *n* values calculated from the Freundlich equation are all in the range of 1.26 to 3.57, implying that the CDM-HCP would be suitable for enriching the adsorption of anilines. The *E*_a_ value of the D–R isothermal adsorption model is the main index to judge whether the adsorption is physisorption or chemisorption. From Table 2, the *E*_a_ values are all greater than 16 kJ/mol, showing that the adsorption process is chemisorption [41].

### 3.5. Thermodynamics of Adsorption

To understand the effect of temperature on the adsorption capacity of the CDM-HCP towards anilines, the results are discussed for the different temperatures. The changes in thermodynamic parameters, including enthalpy change (Δ*H*), entropy change (Δ*S*), and Gibbs free energy change (Δ*G*), were calculated by *K*_L_ at the different temperatures and were fitted based on the isothermal equation and the van ′t Hoff equation. The results are given in the Supplementary Materials. The thermochemical data for the retention of anilines onto the CDM-HCP are listed in Table S2 (Supplementary Materials).

As shown in Table S2 (Supplementary Materials), Δ*H* values were negative, demonstrating that the adsorption process was of an exothermic nature. Moreover, the negative Δ*G* also implied that the adsorption was a spontaneous process. The increase of Δ*G* with temperature increasing indicated that the adsorption of anilines was more beneficial at lower temperatures. Furthermore, the negative Δ*S* showed that the adsorption was an entropy reduction process, which might occur because a large number of anilines were adsorbed onto the adsorbent.

### 3.6. Regeneration Test

The regeneration of adsorbent is crucial for the adsorption process because it can lower the adsorption cost by recovering its adsorption performance from aqueous solutions. Desorption and regeneration experiments were studied for five cycles (Figure S5 (Supplementary Materials)), implying that the desorption process with methanol and HCl solution was efficient, and the saturated absorption capacity can attain 95–99% of virgin CDM-HCP. The results confirmed that the CDM-HCP could be efficiently recovered and it exhibited a good regeneration property. The adsorption capacity of the CDM-HCP was higher than some commercial polymeric adsorbents, such as XAD-4, PA66, and AB-8 (Table S3 (Supplementary Materials)). It showed that the CDM-HCP is a promising adsorbent in the removal of organic pollutants.

### 3.7. Adsorption Mechanism

**Figure 10:**
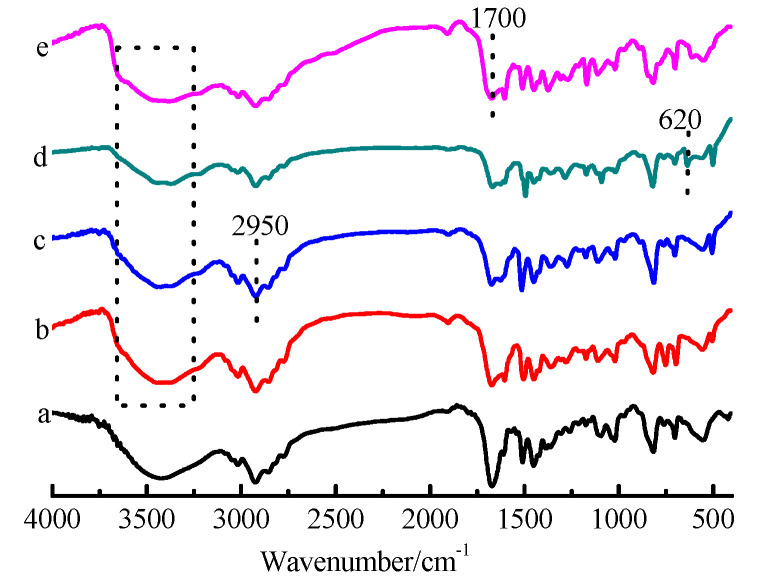
FTIR spectra of the CDM-HCP before and after metal adsorption: CDM-HCP (**a**), CDM-HCP-aniline (**b**), CDM-HCP-*p*-MA (**c**), CDM-HCP-*p*-CA (**d**), and CDM-HCP-*p*-ABC (**e**).

To deduce the adsorption mechanism, FTIR spectra of the CDM-HCP and the CDM-HCP after adsorption of anilines were compared (Figure 10).

**Figure 11:**
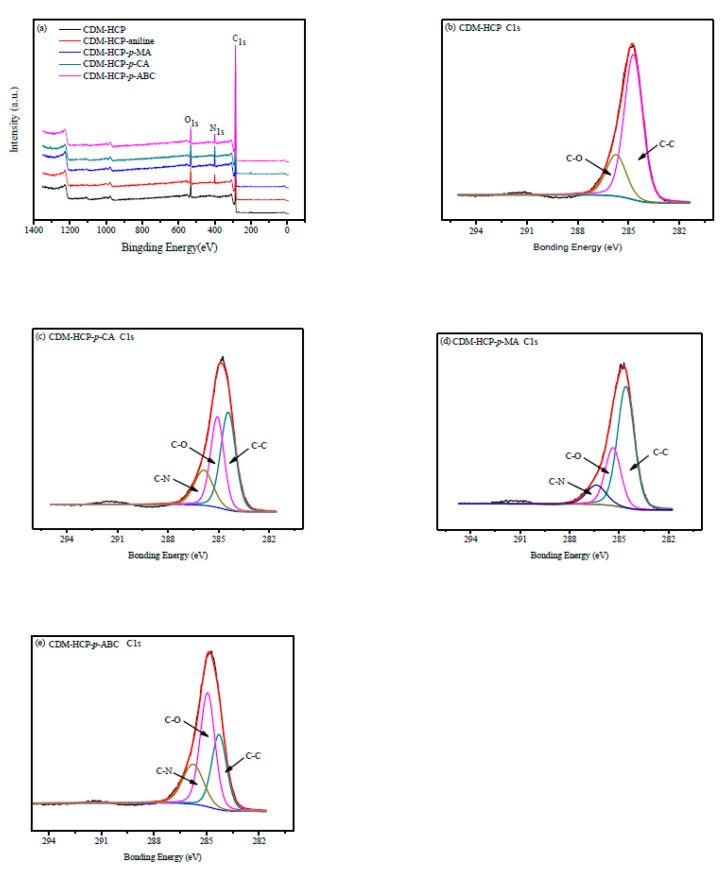
High-resolution XPS spectra of the CDM-HCP (**a**) and C 1s (**b**–**e**) before and after aniline adsorption.

For the FTIR spectra in Figure 10b–e, the broad peak in the region of 3300–3500 cm^−1^ was due to the -OH stretching mode of *β*-CD. The peak intensity weakened slightly and the frequency red-shifted after adsorption of the anilines, implying that there were strong affinities between the CDM-HCP and the anilines. The results demonstrated that the interaction between the functional groups of *β*-CD with the anilines is formed during the adsorption process. To gain further insight into the adsorption mechanism, Figure 11 shows the changes in the composition of the CDM-HCP before and after adsorption.

**Figure 12:**
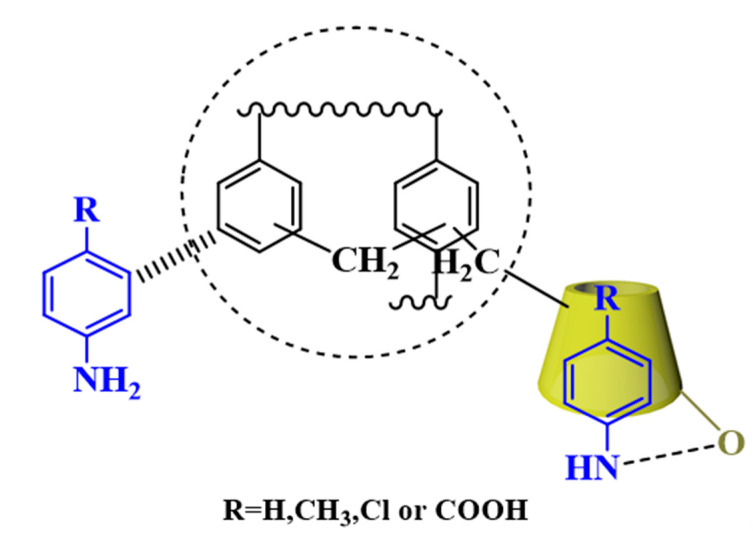
Schematic mechanism of the adsorption of anilines with the CDM-HCP.

As shown in Figure 11, the two peaks around 284.7 eV and 532 eV belong to C1s and O1s, respectively. The new peaks of N1s were observed at 400 eV, which may be attributed to the adsorption by anilines, leading to an increase in nitrogen-containing functional groups. The typical C_1s_ XPS spectra of the CDM-HCP with and without adsorbing anilines are displayed in Figure 11b–e. Before adsorption, the peaks at 284.2 and 285.4 eV for the value states of the CDM-HCP were due to the presence of C = C and C-O, respectively. However, a new peak due to C-N appeared at 286.7 eV, which further proves the adsorption of anilines [35]. The discrete Fourier transform (DFT) method was also used to demonstrate that *β*-CD can form stable complexes with anilines, as shown in Figure S6 (Supplementary Materials). Table S4 (Supplementary Materials) shows the calculated *BE* for *β*-CD/anilines with orientations head down and head up by PM3. The negative binding energies indicated that *β*-CD could interact with anilines to form stable inclusion complexes. The results calculated by ONIOM2 (B3LYP/6-31G(d):PM3) also followed the same trend as those from PM3. The *BE* from the ONIOM2 calculations for the *β*-CD/*p*-ABC head down, *β*-CD/*p*-CA head up, *β*-CD/*p*-MA head up, and *β*-CD/aniline head up were −145.79 ± 2.45, −122.47 ± 1.63, −86.09 ± 1.14, and −78.10 ± 0.89 kJmol^−1^, respectively. They showed that stability of the inclusion complexes followed the sequence of *β*-CD/*p*-ABC > *β*-CD/*p*-CA > *β*-CD/*p*-MA > *β*-CD/aniline, which may lead to the different adsorption capacity. Optimized structures calculated by ONIOM2 also indicated that *β*-CD can form stable complexes with anilines via the O-H⋅⋅⋅O hydrogen bond, as shown in Figure S7 and Table S5 (Supplementary Materials). It was clear that the significantly enhanced adsorption of anilines on the CDM-HCP was mainly driven by hydrogen bonding between adsorbent and adsorbate. Hydrophobic interactions and π–π stacking attractions also played an important role in the aniline adsorption (Figure 12).

## 4. Conclusions

In the present study, a simple and efficient method for the synthesis of a *β*-cyclodextrin-modified hyper-crosslinked resin (CDM-HCP) was presented. It was used to study the removal of anilines from waste effluents. The optimum process, such as amount of *β*-CD in the CDM-HCP, pH, ionic strength, and temperature of the solution was investigated for the improvement of aniline adsorption. The maximum adsorption capacities of the CDM-HCP were nearly 148.97 mg·g^−1^ for aniline, 198.45 mg·g^−1^ for *p*-methylaniline, 293.71 mg·g^−1^ for *p*-chloroaniline, and 622.91 mg·g^−1^ for *p*-aminobenzoic acid, which are higher than some commercial polymeric adsorbents, such as XAD-4, PA66, and AB-8. Aniline adsorption followed the pseudo-second-order kinetic model better. The adsorption isotherms of *p*-MA and *p*-ABC were fitted to the Langmuir model better, whereas aniline and *p*-CA obeyed the Freundlich model better. Moreover, regeneration experiments indicated that the adsorption performance after five cycles nearly attained that of the virgin adsorbent. The interaction mechanism, which was due to the synergistic effects of the weak interactions such as hydrogen bonding, hydrophobic interactions, and π–π stacking attractions, was confirmed by FTIR, XPS, and the theoretical calculation. Briefly, the present adsorption material exhibited great potential for its application in water treatment.

## Figures and Tables

**Table 1:** Kinetic parameters for the adsorption of aniline compounds on the CDM-HCP at 20 °C.

Model	Parameter	Adsorbate			
		Aniline	*p*-MA	*p*-CA	*p-ABC*
this study	*C*_0_ (mg/L)	1000	1000	1000	1000
	*q*_e,expt_ (mg/g)	148.97	198.45	293.71	622.91
pseudo-first-order	*k*_1_ (min^−1^)	4.80 × 10^−3^	5.00 × 10^−3^	4.90 × 10^−3^	2.52 × 10^−3^
	*q*_e,calcd_ (mg/g)	142.61	192.59	291.58	657.69
	*R* ^2^	0.97	0.98	0.97	0.92
pseudo-second-order	*k*_2_ (g/(mg/min))	1.54 × 10^−4^	6.75 × 10^−5^	3.73 × 10^−5^	2.24 × 10^−5^
	*q*_e,calcd_ (mg/g)	130.89	204.50	309.60	684.93
	*R* ^2^	0.99	0.99	0.99	0.99
intraparticle diffusion	*k*_int_ (g/(mg·min^1/2^))	2.18	4.00	7.02	13.92
	*k*_d_	67.85	85.04	99.44	279.07
	*R* ^2^	0.92	0.94	0.96	0.97

**Table 2:** Calculated parameters of Langmuir, Freundlich, and Dubinin–Radushkevich (D–R) Models for the adsorption of anilines.

Model	Temperature/°C	Parameter	Adsorbate			
			Aniline	*p*-MA	*p*-CA	*p-ABC*
Langmuir Model	20	*K*_L_ (L/mg)	9.32 × 10^−3^	6.84 × 10^−3^	1.99 × 10^−3^	1.29 × 10^−2^
		*q*_max_ (mg/g)	163.25	226.44	292.50	753.81
		*R* ^2^	0.968	0.971	0.824	0.995
	25	*K*_L_ (L/mg)	1.31 × 10^−3^	6.74 × 10^−3^	1.98 × 10^−2^	2.67 × 10^−3^
		*q*_max_ (mg/g)	152.93	183.54	254.13	666.40
		*R* ^2^	0.993	0.996	0.838	0.998
	30	*K*_L_ (L/mg)	1.52 × 10^−3^	5.74 × 10^−3^	3.13 × 10^−2^	7.08 × 10^−2^
		*q*_max_ (mg/g)	140.25	171.56	210.18	579.27
		*R* ^2^	0.996	0.997	0.848	0.998
Freundlich Model	20	*K*_f_ (L/mg)	1.71	16.62	48.74	61.42
		*n*	3.03	2.70	3.57	2.50
		*R* ^2^	0.995	0.994	0.982	0.945
	25	*K_f_* (L/mg)	1.95	14.81	40.98	11.89
		*n*	1.58	2.78	3.57	1.56
		*R* ^2^	0.992	0.938	0.989	0.998
	30	*K_f_* (L/mg)	1.90	11.28	22.48	24.67
		*n*	1.64	2.61	4	1.26
		*R* ^2^	0.996	0.959	0.998	0.997
D–R Model	20	*β*	4.22 × 10^−4^	2.89 × 10^−4^	1.41 × 10^−5^	3.99 × 10^−5^
		*q*_m_ (mg/g)	129.87	163.25	240.89	476.56
		*E*_a_ (KJ/mol)	34.44	41.58	91.08	63.09
		*R* ^2^	0.892	0.848	0.808	0.846
	25	*β*	1.15 × 10^−3^	4.84 × 10^−4^	4.36 × 10^−5^	2.15 × 10^−4^
		*q*_m_ (mg/g)	97.46	135.75	210.67	396.24
		*E*_a_ (KJ/mol)	21.45	32.69	78.13	49.47
		*R* ^2^	0.725	0.918	0.737	0.768
	30	*β*	1.35 × 10^−3^	6.26 × 10^−4^	3.93 × 10^−5^	4.15 × 10^−4^
		*q*_m_ (mg/g)	86.59	123.47	186.31	372.94
		*E*_a_ (KJ/mol)	19.74	28.24	64.09	35.88
		*R* ^2^	0.772	0.898	0.766	0.748

## Supplementary Materials

The following are available online at http://www.mdpi.com/2073-4360/12/7/1620/s1/.
