# Supplementary material for: Immobilization of β-CD on a Hyper-Crosslinked Polymer for the Enhanced Removal of Amines from Aqueous Solutions
Source: Polymers (Basel). 2020 Jul 21;12(7):1620. doi: 10.3390/polym12071620 (PMC7408518; doi:10.3390/polym12071620)
Supplement: Supplementary file 1 [file polymers-12-01620-s001.pdf]

Supplementary Information

Immobilization of β-CD on the hyper-cross-linked polymer for the enhanced removal of amines from aqueous solution

Zujin Yang [1,2,*], Guifang Wu [1], Qiuru Li[1], Hongxia, Ai[3], Xingdong Yao[3], and Hongbing Ji [2,3,5,*]

1 School of Chemical Engineering and Technology, Sun Yat-sen University, Zhuhai 519082, China; yangzj@mail.sysu.edu.cn (Z.J.);1244642515@qq.com (G. F.); 1045757624@qq.com (Q. R.)
2 School of Chemical Engineering, Huizhou Research Institute of Sun Yat-sen University, Huizhou 516216, China
3 Fine Chemical Industry Research Institute, The Key Laboratory of Low-carbon Chemistry & Energy Conservation of Guangdong Province, School of Chemistry, Sun Yat-sen University, Guangzhou 510275, China
4 The Key laboratory of Forest Chemistry & Engineering of Guangxi, Guangxi University for Nationalities,Nanning, China; 271837377@qq.com
5 School of Chemical Engineering, Guangdong University of Petrochemical Technology, Maoming 525000, China
* Correspondence: jihb@mail.sysu.edu.cn (H.J.)

Academic Editor: Editor: Prof. Dr. Francesco Paolo La Mantia

Received: June 22, 2020

Computational methods

All theoretical calculations were carried out with Gaussian 09 software package [1]. The initial structure of aniline, *p*-MA, *p*-CA, or *p*-ABC was constructed using the CS Chem3D (version 14.0) program. Initial β-CD coordinates were obtained from crystallographic data [2, 3]. In order to simulate the inclusion process, the glycosidic oxygen atoms of β-CD were placed onto the *XY* plane and their center was defined as the center of the coordination system. The glycosidic oxygen atoms of β-CD were placed onto the XY plane and their center was defined as the center of the Cartesian coordination system. The primary hydroxyl groups of β-CD were pointed toward the positive direction of **Z**-axis. The inclusion complex was constructed from the PM3-optomized β-CD and the corresponding guest molecule. The longer dimension of the guest molecule was initially placed onto the *Z*-axis, and the relative position between the host and the guest molecule was measured by the *Z*-coordinate of the labeled carbon atom of the guest as shown in Fig. 2. The inclusion complex was emulated by entering guest molecule from one end of β-CD and letting it passing through β-CD by changing Z_{C^*} from +8 to -8 (head up) or -8 to +8 (head down) at 1 interval, and by scanning θ from 0° to 360° at 20° intervals. Each complex was minimized in energy under vacuum. The lowest energy complex was used as the starting geometries for single point calculation and ONIOM analysis, and the geometry structure for every point was also optimized by PM3 method without any restriction. DFT single point calculations at the level of B3LYP/6-31G were performed for the PM3-optimized complex in vacuo. The binding energy (*BE*) could be expressed by *E*q. (1):

$$BE = E_{\text{complex}} - E_{\text{host}} - E_{\text{guest}} \tag{1}$$

E_{complex} was the total energy of complex, E_{guest} was the sum of total energy of guest, and E_{host} was the total energy of host.

Adsorption Model Fitting

Kinetic models have been used to understand the adsorption mechanism and test performance of the adsorbent for the adsorption of anilines. Pseudo-first-order, pseudo-second-order and intraparticle diffusion models were used to interpret the adsorption data of the anilines by CDM-HCP.

(a) The pseudo-first-order kinetic equation is represented by

$$\ln\left(q_e - q_t\right) = \ln q_e - k_1 t \tag{2}$$

where q_t (mg·g^{-1}) is the amount of each aniline adsorbed at time *t* (min), q_e (mg/g) the equilibrium adsorption capacity, and k_1(L·min^{-1}) the rate constant.

(b) The pseudo-second order model can be expressed by

$$\frac{t}{q_t} = \frac{1}{K_2 q_e^{\,2}} + \frac{t}{q_e} \tag{3}$$

where q_t (mg·g^{-1}) is the amount of each aniline adsorbed at time *t* (min), q_e (mg/g) the equilibrium adsorption capacity, and K_2 (g/(mg·min)) the constant rate.

(c) Since neither the pseudo first-order nor the second-order model can identify the diffusion mechanism, the intra-particle diffusion model is used to elucidate the diffusion mechanism, which model can be described by,

$$q_t = k_i t^{1/2} + C \tag{4}$$

where k_i is the intraparticle diffusion constant (mg/(g·min$^{1/2}$)). The values of C and k_i are calculated from the intercept and slope of the linear plot of qt versus $t^{1/2}$, respectively.

To determine the mechanism of the adsorbent, Langmuir, Freundlich, and Dubinin-Radushkevich (D-R) models were extensively applied for the removal of pollutants from water [4].

The Langmuir equation can be defined,

$$\frac{c_e}{q_e} = \frac{c_e}{q_m} + \frac{1}{k_L q_m} \tag{5}$$

where c_e (mg·L^{-1}) and q_e (mg·g^{-1}) are the concentration of adsorbate and adsorption capacity of the adsorbent at equilibrium time, respectively. K_L is Langmuir adsorption constant and q_m (mg·g^{-1}) is the theoretical maximum adsorption capacity to form monolayer on the resin, respectively.

The Freundlich isotherm is used to describe multilayer adsorption with interaction between the adsorbed molecules, and is determined as,

$$\ln q_e = \ln K_f + \frac{1}{n} \ln c_e \tag{6}$$

where K_f is Freundlich constant that is an indicator of adsorption capacity and $1/n$ is the Freundlich coefficient related to the magnitude of the adsorption driving force.

To further understand of adsorption mechanism, the Dubinin-Radushkevich isotherm (D-R) is used to describe the adsorption process of pollutants as physical or chemical adsorption. Its linear form can be represented,

$$\ln q_e = \ln q_m - \beta \varepsilon^2 \tag{7}$$

where q_m is the theoretical saturation capacity (mg/g), β is related to mean adsorption energy (kJ/mol) , and ε(Polanyi potential) is calculated as,

$$\varepsilon = RT \ln\left(1 + \frac{1}{c_e}\right) \tag{8}$$

R (kJ/(mol·K)) is the gas constant and T (K) is absolute temperature. q_m and β are obtained from the intercept and the slope of linear plot of ln q_e against ε^2, respectively. E_a (kJ/mol) is the mean adsorption energy, which is calculated from the β value as:

$$E_a = \frac{1}{(2\beta)^{1/2}} \tag{9}$$

If E_a is > 16 kJ/mol, the adsorption process is a chemisorption, and it is a process of ion exchange when E_a is between 8 and 16 kJ·mol^{-1}, while for values of E_a < 8 kJ·mol^{-1}, it is a physical process [5].

To understand the effect of temperature on the anilines adsorption on CDM-HCP, enthalpy change (△H), entropy change (△S), and Gibbs free energy change (△G) were determined by the values of K_L at the different temperatures were fitted according to the isothermal equation [*Eq.* (10)] and the van't Hoff equation [*Eq.* (11)]:

$$\Delta G = -RT \ln K_L \tag{10}$$

$$\ln K_L = -\frac{\Delta H}{RT} + \frac{\Delta S}{R} \tag{11}$$

where R (8.314 J/(mol·K)) is the gas constant, T(K) is the absolute temperature, and K_L (L·mol^{-1}) is the Langmuir constant. ΔH and ΔS are calculated from the slope and the intercept of a linear plot of ln K_L against 1/T.

Figures and Tables

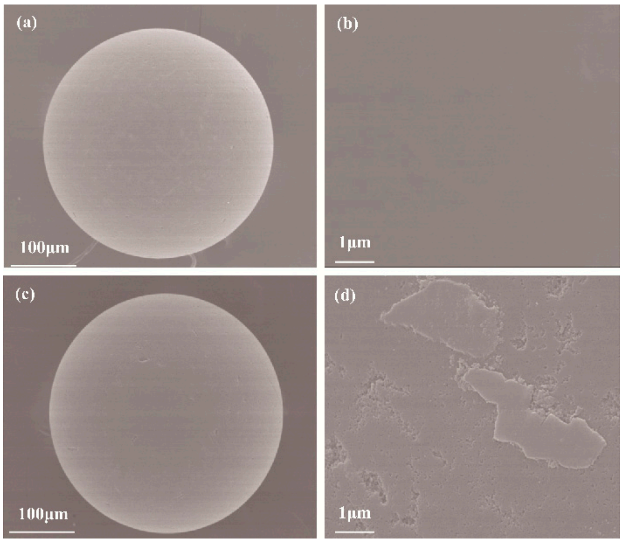

Figure S1. SEM of CM-PS (a-b), and CDM-HCP (c-d).

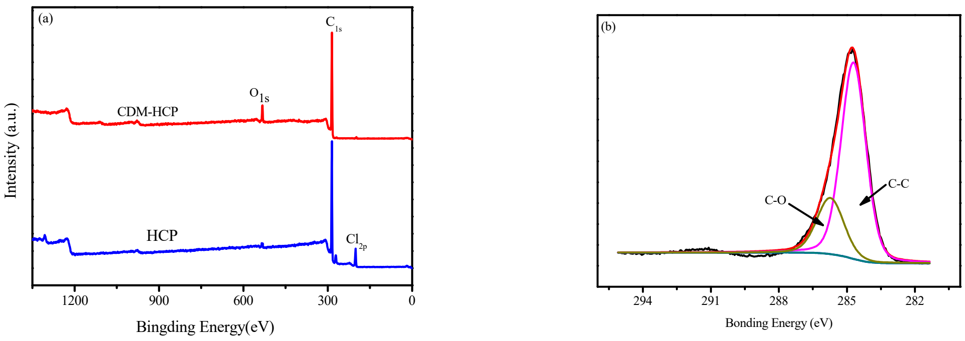

Figure S2. XPS wide scan of HCP (a-b), and high-resolution XPS spectra of CDM-HCP (b).

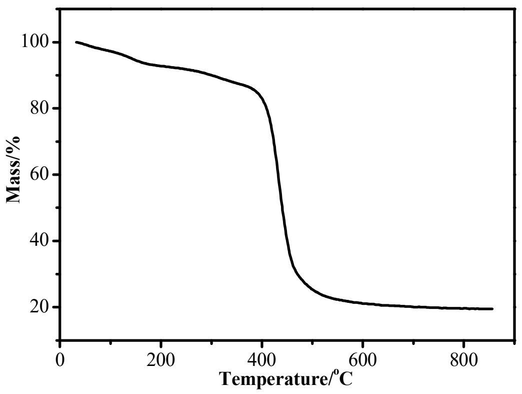

Figure S3. TG of CDM-HCP.

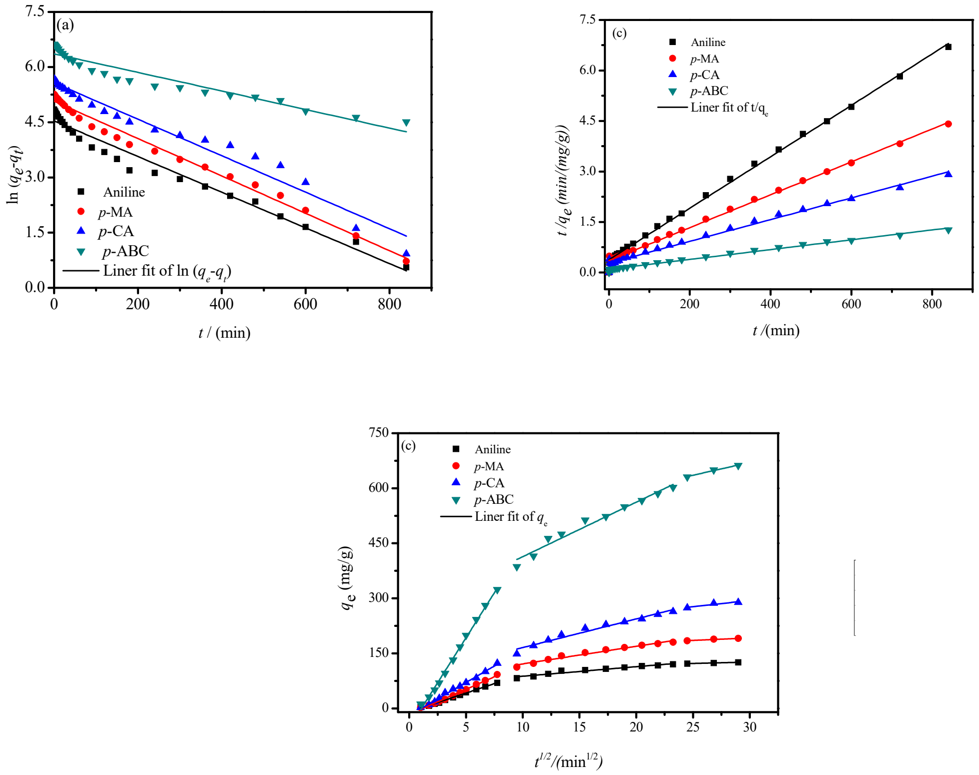

Figure S4. Fitting of adsorption kinetic equations of anilines onto CDM-HCP. (a) Pseudo-first-order kinetics ; (b)Pseudo-second-order kinetics; (c) Intraparticle diffusion kinetics.

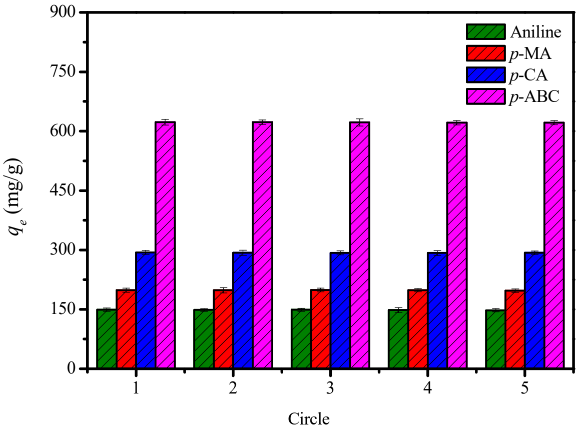

Figure S5. Regeneration cycle on the adsorption capacity of anilines on CDM-HCP with adsorption capacity determined within an error of less than 5%.

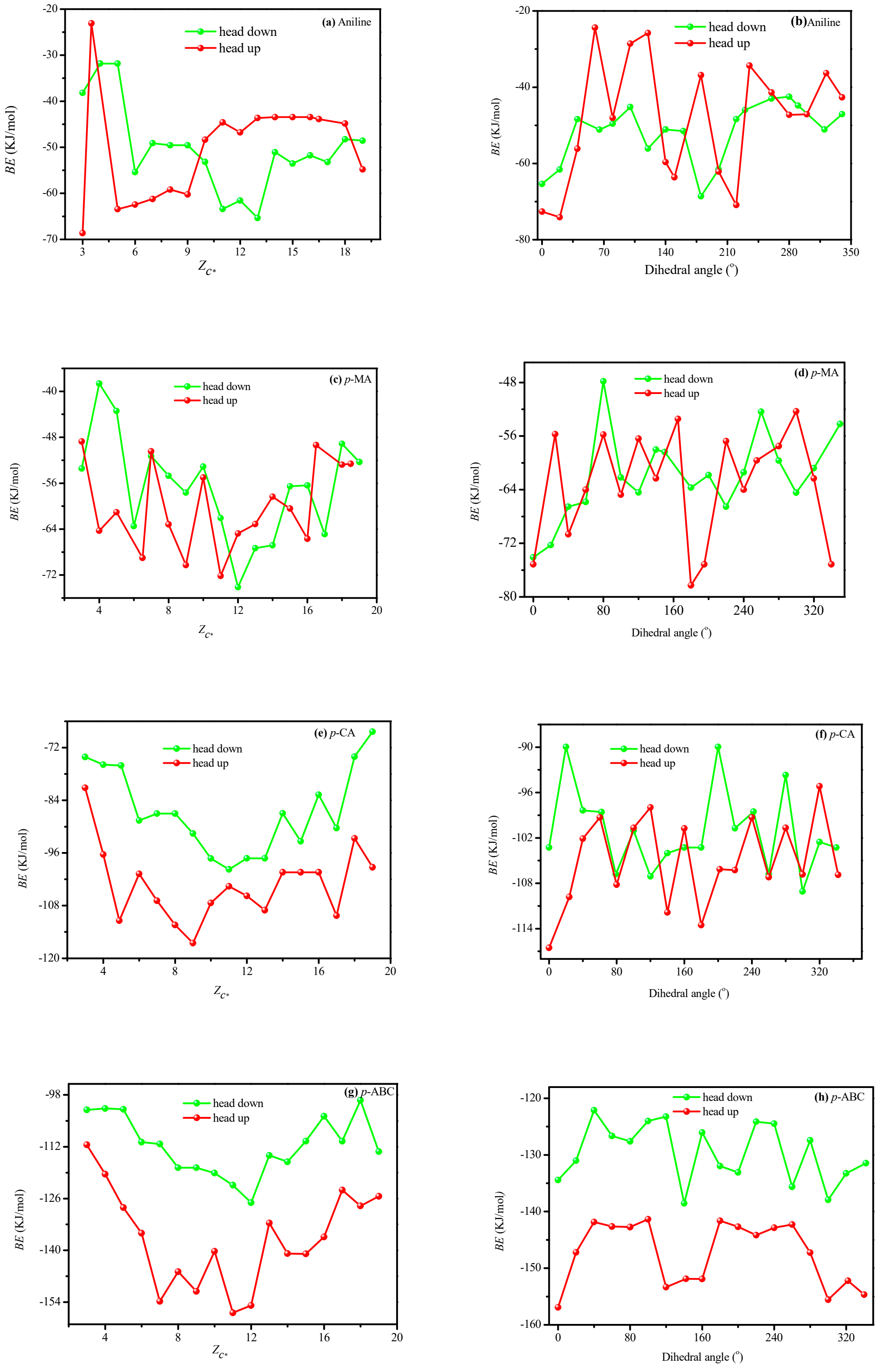

Figure S6. PM3 calculated binding energy of β-CD with aniline, *p*-MA, *p*-CA, and *p*-ABC at different positions (Z_{c*}), a, c, e, g; angle(θ), b, d, f, h with *BE* determined within an error of less than 5% .

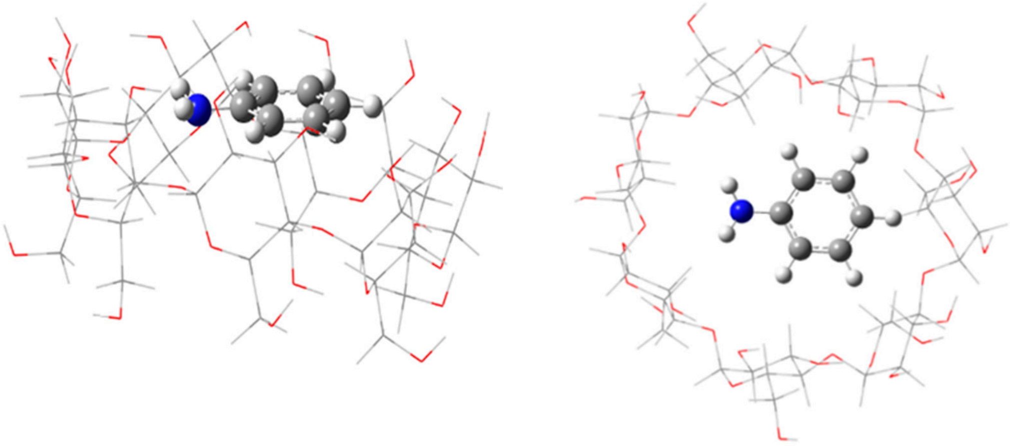

(a) *β*-CD/aniline BE_2 = -78.10 ± 0.89 kJ/mol

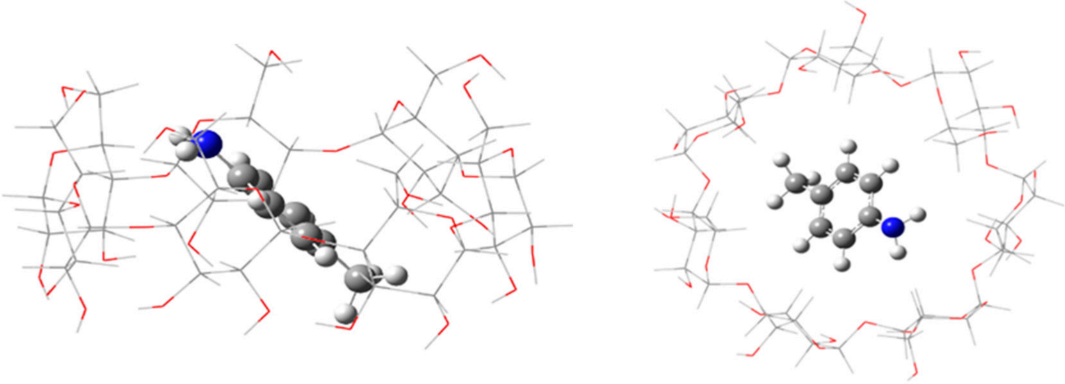

(b) *β*-CD/*p*-MA BE_2 = -86.09 ± 1.14 kJ/mol

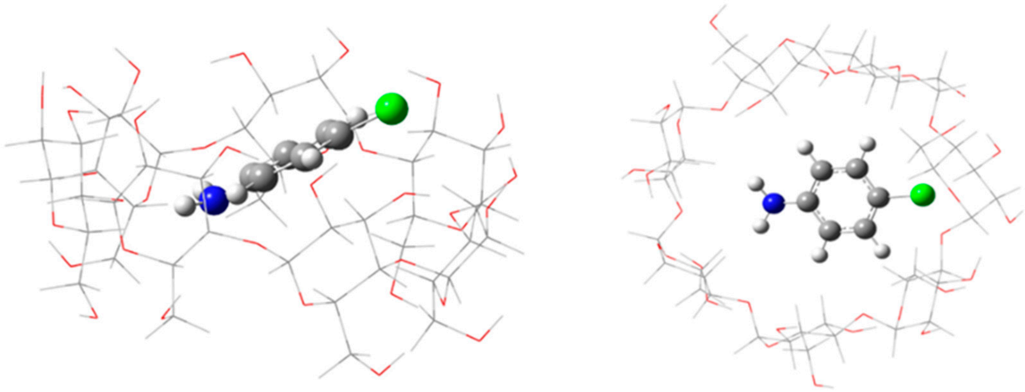

(c) *β*-CD/*p*-CA BE_2 = -122.47 ± 1.63 kJ/mol

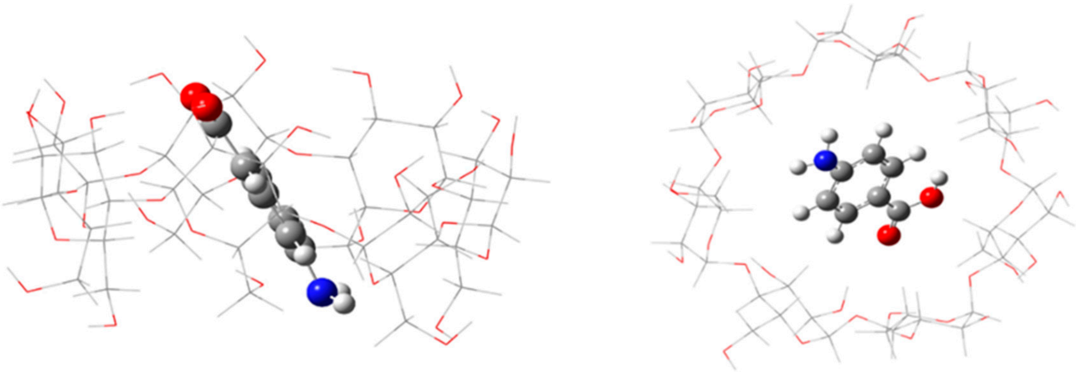

(d) *β*-CD/*p*-ABC *BE*$_2$ = -145.79 ± 2.45 kJ/mol

Figure S7. The structure of *β*-CD complexes with (a) aniline, (b) *p*-MA, (c) *p*-CA, and (d) *p*-ABC by ONIOM2 (B3LYP/6-31G(d):PM3).

Table S1. BET characterization of CM-PS, HCP, and CDM-HCP

Materials	Surface area(m^2/g)		Pore volume (cm^3/g)	Average pore size (nm)	Chlorine content (%)	O content (%)	Contact angle (°)
	S_{BET}	S_{micro}					
CM-PS	44.38	3.67	0.06534	0.78	18.04	0	137
HCP	320.90	168.34	2.3430	75.32	4.32	0	141
CDM-HCP	194.52	46.77	0.5120	33.49	0.24	6.79	108

Table S2. Thermodynamic parameters for adsorption of anilines on CDM-HCP

Adsorbate	Temperature/ K	△*G* (KJ/mol)	△*H* (KJ/mol)	△*S* (J/(mol·K))
	293.15	-7.39		-132.38
Aniline	298.15	-3.93	-46.19	-141.74
	303.15	-4.13		-138.75
	293.15	-6.59		-158.42
p-MA	298.15	-6.89	-53.03	-154.76
	303.15	-6.63		-153.04
	293.15	-8.70		-211.85
p-CA	298.15	-8.85	-70.81	-207.80
	303.15	-10.08		-200.32
	293.15	-6.09		-452.86
P-ABC	298.15	-3.87	-138.85	-452.72
	303.15	-3.19		-447.50

Table S3. Comparisons in anilines adsorption of various adsorbents

Adsorbent	Adsorbate (mg/g)			
	aniline	*p*-MA	*p*-CA	*p*-ABC
CDM-HCP	148.97	198.45	293.71	622.91
XAD-4	138.35	1781.88	239.11	345.03
PA66	116.27	181.91	263.83	225.79
AB-8	105.18	156.12	286.68	204.23

Table S4. Binding Energy of β-CD complexes with aniline, *p*-MA, *p*-CA, and *p*-ABC calculated by PM3 and ONIOM2 (B3LYP/6-31G(d):PM3)

Inclusion complexes	Orientation	Z^*_C(A)	BE_2 (kJ/mol)	θ(°)	BE_2 (kJ/mol)	ONIOM2 BE_2(kJ/mol)
β-CD/aniline	Head down	13	-65.35	180	-68.59	-70.69
	Head up	3	-68.64	20	-74.09	-78.10
β-CD/*p*-MA	Head down	12	-74.11	0	-74.11	-81.57
	Head up	11	-72.14	180	-78.30	-86.09
β-CD/*p*-CA	Head down	11	-99.72	300	-109.08	-115.08
	Head up	9	-116.54	0	-116.54	-122.47
β-CD/*p*-ABC	Head down	12	-127.12	140	-138.52	-139.74
	Head up	11	-156.92	0	-156.92	-145.79

Table S5. Number, type, bond length (*r*), bond angle (*A*) and *BE* of intermolecular hydrogen bond between hydrates and *β*-CD calculated by ONIOM2 (B3LYP/6-31G(d):PM3)

Inclusion complexes	Number	Type	*r* (Å)	*A* (°)	BE_2(kJ/mol)
β-CD/aniline	1	N-H…O	2.832	103.66	-78.10
β-CD/*p*-MA	1	N-H…O	2.623	126.39	-86.09
β-CD/*p*-CA	1	N-H…O	2.112	109.96	-122.47
β-CD/*p*-ABC	1	N-H…O	1.769	111.07	-145.79

References

1. Frisch, M.; Trucks, G.; Schlegel, W. H. B. Gaussian 98. Revision A 7. *Gaussian Inc Pittsburgh PA* **1998**.
2. Lindner, K.; W, Saenger. Crystal and molecular structures of cyclomaltoheptaose inclusion complexes with HI and with methanol. *Carbohydrate. Res.* **1982**, *107*, 7-16.
3. Stewart, J. J. P. Optimization of parameters for semiempirical methods I. Method. *J. Comput. Chem.* **1989**, *10*, 221-264.
4. Chen, Y. W.; Wang, J. L. Preparation and characterization of magnetic chitosan nanoparticles and its application for Cu(II) removal. *Chem. Eng. J.* **2011**, *168*, 286-292.
5. Fu, Y. J.; Zu, Y. G.; Liu, W.; Hou, C. L.; Chen, L. Y.; Li, S. M.; Shi, X. G.; Tong, M.H. Preparative separation of vitexin and isovitexin from pigeonpea extracts with macroporous resins. *J. Chromatogr. A.* **2007**, *1139*, 206-213.
